# An Impact of Psychological Stress on the Interplay between Salivary Oxidative Stress and the Classic Psychological Stress-Related Parameters

**DOI:** 10.1155/2021/6635310

**Published:** 2021-01-07

**Authors:** Nikola M. Stojanović, Pavle J. Randjelović, Dragana Pavlović, Nenad I. Stojiljković, Ivan Jovanović, Dušan Sokolović, Niko S. Radulović

**Affiliations:** ^1^Department of Physiology, Faculty of Medicine, University of Niš, Zorana Đinđića, 81 Niš, Serbia; ^2^Department of Pharmacy, Faculty of Medicine, University of Niš, Zorana Đinđića, 81 Niš, Serbia; ^3^Department of Anatomy, Faculty of Medicine, University of Niš, Zorana Đinđića, 81 Niš, Serbia; ^4^Department of Biochemistry, Faculty of Medicine, University of Niš, Zorana Đinđića, 81 Niš, Serbia; ^5^Department of Chemistry, Faculty of Sciences and Mathematics, University of Niš, Višegradska, 33 Niš, Serbia

## Abstract

Both oxidative and psychological (mental) stress are the likely culprits for several acute and chronic health disturbances, and adequate tests mimicking that are needed. Herein, in controlled laboratory surroundings, a PEBL (Psychology Experiment Building Language) test battery was used to evoke stress-related biological responses followed by tracking changes in saliva parameters. The study objectives were to determine the impact of psychological stress on selected salivatory parameters and to assess the correlation between the determined oxidative and stress parameters. The study was conducted on 36 healthy young subjects, mainly females (*n* = 24). Before and following the completion of a battery of four PEBL tests, subjects' saliva samples were collected. Stress-evoking changes in total antioxidant capacity and nitrite/nitrate levels, as oxidative stress parameters, and cortisol and immunoglobulin A (IgA), as parameters of psychological stress, were established and mutually correlated by comparing the values of the evaluated parameters pre- and post-PEBL test. The results showed that there is no change in the total salivary antioxidant capacity (*p* > 0.05); however, there was a significant increase in nitrites/nitrates levels after the PEBL test (*p* = 0.007). On the other hand, the determined cortisol levels after the test battery were found to be statistically significantly increased (*p* = 0.025) when compared to the values obtained before the test, while the levels of IgA were found to be statistically significantly decreased (*p* < 0.001). The only statistically significant correlation between the changes in the studied parameters was found to be the one between cortisol and IgA levels (Spearman's Rö = -0.4). These results suggest that the short-term stress induced by the PEBL test does evoke changes in the salivary mental stress-related parameters (an increase in cortisol and nitrite/nitrate levels, and a decrease in IgA), but not in the total antioxidant capacity. They also indicate that the constructed PEBL four-test battery might represent an adequate laboratory stress-inducing paradigm.

## 1. Introduction

In the modern world, stress is believed to be a part of everyday life and might be regarded as a culprit for a great number of acute and chronic health disturbances [1]. There are numerous definitions of stress, and stress has a different meaning for different people under different conditions [2]. The first and broadest definition is the one given by Hans Selye, the “father of stress”, who says “Stress is the non-specific response of the body to any demand”. In behavioral sciences, stress is regarded as the “perception of threat, with resulting anxiety discomfort, emotional tension, and difficulty in adjustment,” while in terms of neuroendocrinology, stress can be defined as any stimulus that provokes a release of cortisol [2]. Regardless of what the definition of stress is, no one can overlook the immense impact of stress on human health and daily functioning [1]. Mild stress can be quite useful since it may enhance task and cognitive performance; on the other hand, constant and high-intensity stress leads to anxiety and depression [3].

During the past decades, a link was found between stress and common biological pathways which in turn affect basic physiological and pathological processes in vertebrates [1]. An integral part of the response to stress includes the activation of stress neural circuits, which link several brain regions involved in basic sensory and motor functions for perception and motor response to a stressful challenge, respectively, as well as more intricate autonomic, neuroendocrine, cognitive, and behavioral activities [4]. Major roles are played by the autonomic nervous system and the hypothalamic-pituitary-adrenocortical axis (HPAA) which are acting as afferent and efferent signaling pathways connecting the stress impute with the rest of the body [2]. Initial exposure to stressor activates the alarm reaction resulting in sympathetic discharge and an increased secretion of glucocorticoids [2, 5]. These biological processes, happening after exposure to stress, enabled the “measurement” of stress through cortisol concentrations [2]. A recent study pointed to the potential significance of salivary immunoglobulin A (IgA) as the useful biomarker of stress [6].

Every biological fluid, including saliva, contains different antioxidant mechanisms which at any point are ready to cope with a stressor of a different etiology. The syntagm “the mirror of health of the organism,” used to describe saliva, explains just how good saliva is as a source of body biological response parameters to psychological stress [7]. Apart from that, the saliva represents a rapidly changing dynamic environment, and its collection is comfortable, noninvasive, and nonexpensive compared to other biological sources [8]. Saliva proteins and other metabolites are synthesized/modified/excreted by the major salivary gland cells (the parotid, submandibular, and sublingual glands), the function of which is most frequently depending on *β* adrenergic stimulation [9]. Oxidants that can induce macromolecule damage mainly arise from activation of the mitochondrial respiratory chain (superoxide radical), and also the cellular metabolism of non-stressed cells [10]. Thus, it is not surprising that oxidative damage is related to disease incidence, severity, morbidity, and mortality [11]. It is suggested that free radicals generated by the catecholamine autooxidation contribute to tissue oxidative damage associated with psychosocial stress [12]. Interestingly, some studies suggest that psychological stress can sometimes cause intensified resilience of tissue to oxidative stress [11, 13]. The antioxidant capacity is comprised of an enzymatic and nonenzymatic (low molecular) component which are trying to balance and prevent an increase in oxidants (reactive oxygen/nitrogen species) which can lead to oxidative damage [14].

Although there are protocols designed to induce stress, such as psychological stress, physical exercise, pharmacological stimulation, real-life stressors, and laboratory settings, each of them has some downfalls, as well as certain advantages [15]. The Psychology Experiment Building Language (PEBL) represents a free software package [16] that enables the construction of a computerized test battery that can aid in the estimation of cognitive capabilities that involve memory, attention, and executive control [17]. The design of PEBL is such that it allows one to construct a test battery (“experiment chains”), while each task in the chain can be considered a stand-alone examination [17]. The usage of PEBL is wide-spread in disciplines such as cognitive psychology [18], neurology [19], behavioral endocrinology [20], neuropharmacology [21], physiology [21], etc.

Having in mind that there is a limited number of reliable laboratory stress-inducing tests that can evoke a proper biological response through HPAA, this study aims at constructing one. Also, we aimed to determine if the same stress-inducing test can induce changes in the total salivary antioxidant status. Using a PEBL test battery consisting of 4 tasks, we deemed to acutely provoke stress and biological response in healthy young subjects. Stress-evoking changes in biological parameters, cortisol, IgA, the total antioxidant activity, and NO levels, would be determined in the subjects' saliva by comparing the values of the evaluated parameters pre- and post-PEBL test. Finally, the obtained changes in stress-related parameters (cortisol and IgA) would be correlated with the ones obtained for salivary oxidative status (the total antioxidant activity and NO levels). Thus, the objectives were to determine the impact of psychological stress on selected salivatory parameters and to assess the correlation between the determined oxidative and stress parameters.

## 2. Materials and Methods

### 2.1. Subjects

**Figure 1:**
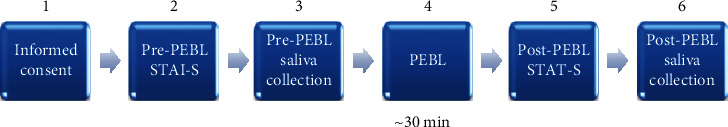
A schematic representation of the experimental procedure summary. STAI-S, Spielberger's State-Trait Anxiety Inventory-State; PEBL, Psychology Experiment Building Language.

Twelve male and twenty-four female subjects (volunteers), aged from 20 to 23, took part in this study (the study group characteristics are given in Table 1), which was previously approved by the Ethics Committee of the Faculty of Medicine, the University of Niš, Serbia (No. 12-6316-2/11). The study was conducted according to the standards given in the Declaration of Helsinki (revised in 2008). Prior to participation, each volunteer signed an informed consent and completed a medical health questionnaire that enabled its inclusion. All subjects were reported to be healthy (no acute or chronic diseases), drug- and medication-free, with the exception of taking contraceptive pills (for one female), and had no known food/drug allergies. Smokers and chronic alcohol consumers were not included in the study. Participants did not consume caffeine-containing products or alcohol for a minimum of 24 h prior to the testing. On the morning of the test, subjects were instructed to avoid eating breakfast.  Figure 1 represents a schematic of the summary of the experimental procedure.

### 2.2. Study and PEBL Battery Design

The study was conducted at a computer laboratory at the Department of Physiology, Faculty of Medicine, University of Niš. The subjects were tested on three consecutive days between 8 and 10 AM, and all participants were randomly divided into three groups of 12.

The test battery consisted of the following tasks:

- *Corsi blocks test*. The subjects were presented with a screen containing nine dark squares and each square lit up in a sequence. The subjects were instructed to remember the order in which the squares were lit, and after the sequence was completed, they were to select the squares in the order they lit up [22]. Each task took approximately 5 minutes
- *Forward digit span*. The subjects were presented with a series of numbers, which, after every correct answer, increased in its length, and were required to immediately repeat them back. Longer series of numbers were presented to subjects until they could no longer repeat them back correctly [22]. The task took five minutes to complete
- *Speeded tapping/oscillation task*. Subjects were instructed to tap the space button on a keyboard with their dominant hand index-finger for as many times as they could during 60 secs. After each test period, the results for the previous test were shown on the screen during the 10 sec break period. The test consisted of 3 consecutive sessions
- *Simple response time*. Subjects were presented with a signal (letter X) following an irregular time pattern (250 to 2500 ms) and were instructed to react (press a button on the keyboard) as soon as they see the stimulus. The test was comprised of 4 blocks of 50 trials and it took around 6 min to finish.

### 2.3. Psychological Stress Level Determination

The extent of stress induction was estimated based on Spielberger's State-Trait Anxiety Inventory (STAI) which consists of state (S) and trait (T) scales each consisting of 20 4-point Likert scale questions [23]. Each of the two scales contained two factors, which were labeled as anxiety present and anxiety-absent factors. The scale was translated from English to Serbian and then back-translated into English, with a Cronbach alpha of 0.713 and 0.858 for S- and T-scale, respectively [23]. The S-anxiety scale which measures immediate feelings of anxiety was only used in the current work.

### 2.4. Sample Collection and Protein Determination

Saliva samples were collected 15 min before and immediately after the PEBL test. The subjects enrolled in the study were instructed not to consume alcohol at least 24 h prior to the test. Also, on the morning of the test, subjects avoided food intake. Saliva samples were collected from subjects using a passive method which consisted of passive drooling into a 10 mL sterile polystyrene tubes with round bottoms (16.8 x 82 mm; Sarstedt®, Germany) for five minutes [24]. After collection, samples were refrigerated for no longer than 15 min and centrifuged at 3000 g for 15 min (4°C) in order to obtain a clear supernatant that was further used. All samples were divided into four parts and frozen at -80°C in different sterile tubes for different analyses in order to avoid multiple freezing and thawing procedures. In all cases, the volume of saliva obtained after centrifugation was no less than 1 mL.

Protein content was determined based on a standard Lowry's method [25] without any changes. The amount of saliva protein was calculated based on values obtained from a bovine serum albumin standard curve.

### 2.5. Biochemical Analyses

#### 2.5.1. Total Salivary Antioxidative Capacity Determination

The free radical scavenging activities of the centrifuged saliva samples were determined using the stable 1,1-diphenyl-2-picrylhydrazyl (DPPH) free radical [26]. When reduced to DPPH-H, the color changes from deep violet to yellow and is measured spectrophotometrically against a blank at 517 nm or according to our slight modifications on an ELISA microplate reader at 540 nm [27]. Forty microliters of each sample were mixed with 120 *μ*L of methanol and 40 *μ*L of methanolic DPPH solution (0.05 mM) in each of the wells of the 96-well microtiter plate. The mixture was shaken briefly and left in the dark at room temperature for 30 min, and the absorbance was determined using an ELISA reader. Quenching of the DPPH free radical, in percent, was calculated according to the following equation:

(1) $$\begin{array}{c} \%\text{DPPH}=\left(\text{Ac}–\frac{\text{As}}{\text{Ac}}\right), \end{array}$$

where Ac is the absorbance of the control reaction (containing methanol instead of the test sample) and As is the absorbance of the saliva sample. The results are expressed as differences in % of quenching for paired samples (the saliva from the same subject after and before the test procedure). The assays were carried out in triplicates.

#### 2.5.2. Salivary Cortisol Determination

Cortisol levels in the collected saliva samples were determined using the ELISA kit (R&D Systems, USA) following the manufacturer's protocol. The assay standard curve range was from 0.156 to 10 ng/mL, where the standard curve was highly reproducible (mean *R*^2^ = 0.997). All samples were assayed in duplicate and their average was used in further analyses.

#### 2.5.3. Salivary IgA Determination

Levels of salivary IgA were determined using eBioscience ELISA kit (Human IgA ELISA Ready-SetGO!; Cat.No. 88-50600), and the procedure was performed following the manufacturer's protocol. The assay standard curve range was from 1.6 to 100 ng/mL.

#### 2.5.4. Salivary NO_2_^−^/NO_3_^−^ Determination

The concentration of nitrites/nitrates present in the saliva was determined in mixtures consisting of equal volumes (100 *μ*L) of saliva sample and the Griess reagent. The absorbance was measured at 540 nm using a microplate reader (Multiskan Ascent No. 354, Thermo Labsystems, Finland), and the concentrations (*μ*mol/L) were calculated using a standard curve of sodium nitrite.

### 2.6. Statistical Analysis

Sample size, calculated using the G-Power software with the value of *α* = 0.05, strength of the study set at 0.8, while the effect size was set at 0.6, was found to be >30. The obtained results from the biochemical measurements were given as median values. Additionally, minimal (MIN) and maximal (MAX) values are given. In order to determine the data distribution, the Shapiro-Wilk test was used. The differences between the pre- and posttest values, which did not show normal distribution, were compared using the Wilcoxon signed ranks test (SPSS 23, IBM), and the *p* values less than 0.05 were considered statistically significant. Effect size estimate (*r*) was calculated based on the *z* values obtained from the Wilcoxon signed ranks test. The effect size estimate (*r*) values were interpreted as follows: >0.1, small; >0.3, medium; and >0.5, large [28]. The magnitude of the correlation between the changes in the studied biochemical parameters was expressed as Spearman's Rö and *p* values. The obtained coefficients were treated as follows: negligible (0.0-0.1), weak (0.1-0.2), moderate (0.2-0.4), relatively strong (0.4-0.6), strong (0.6-0.8), and very strong (>0.8) [28].

## 3. Results

**Figure 2:**
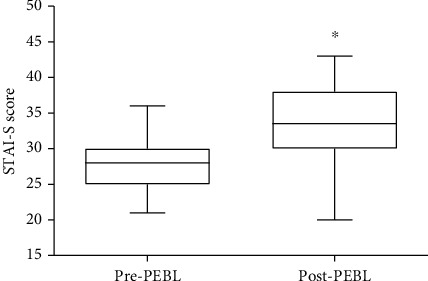
Boxplots showing lower (Q1) and upper (Q3) quartiles, as well as median values for STAI scores obtained pre- and post-PEBL test. Data comparison (*n* = 36) was done using the Wilcoxon signed ranks test, ∗*p* < 0.001 vs. pre-PEBL score with *z* = −4.804.

The scores obtained for the STAI-S scale pre- and post-PEBL test did not show normal distribution; thus, the data were compared using the Wilcoxon signed ranks test. The lower (Q1) and upper (Q3) quartiles for STAI-S scale pre- and post-PEBL test were 25 and 30, and 30 and 38, respectively (Figure 2). On the other hand, the median for STAI-S scale pre- and post-PEBL tests were 28 and 34, respectively (Figure 2). When the scores of STAI-S were compared, the difference between pre- and post-PEBL test scores was found to be statistically significant (Figure 2).

The normality test did not show a normal distribution pattern; thus, for the comparison between the values of different biochemical parameters obtained pre- and post-PEBL test, we used the Wilcoxon signed ranks test. Salivary oxidative capacity, determined using the DPPH assay, revealed that there is a significant change in the oxidative capacity induced by the PEBL test. Cortisol salivary levels were found to be statistically significantly increased after the PEBL test battery with moderate effect size, while the levels of IgA were found to be statistically significantly decreased, with strong effect size, after the test (Table 2). In addition, we found that the estimated salivary concentrations of NO_2_^−^/NO_3_^−^ were statistically significantly increased after the PEBL test battery, with a moderate effect size (Table 2). The calculated percentage change between pre- and post-PEBL test determination for oxidative capacity, cortisol, IgA, and NO_2_^−^/NO_3_^−^ were 0.9, 52.9, -37.9, and 61.1%, respectively.

**Figure 3:**
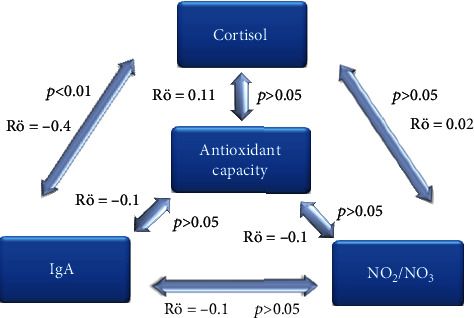
Schematic representation of the correlation coefficients (Spearman's Rö) and *p* values between the studied oxidative and mental stress-related parameters.

The correlation analysis was done with a percentage change in the studied parameter, and not the absolute value, in order to exclude the potential influence of exogenous molecules on antioxidant status and NO concentrations. Since these data did not show normal distribution, we applied a method of Spearman's correlation. Some degree of weak correlation (Rö < 0.2) between the values of total antioxidative capacity and NO concentrations and both psychological stress-related parameters (Figure 3) was determined; however, that correlation was found not to be statistically significant (*p* < 0.05). The only significant correlation (negative) was found between the changes in cortisol and IgA levels (Figure 3).

## 4. Discussion

Extensive research in the field of psychoneuroendocrinology/-immunology is still struggling to find the answer whether, and to what extent, the physiological stimulus (stress) is connected to a biological response. The design of this study aimed at fulfilling some criteria in order to possibly avoid the most common methodological issues [15]. The sample chosen for the study was uniform and consisted of healthy young subjects of similar age. In an attempt to induce central stress (directly at the level of the central nervous system), we designed a test conducted in a laboratory environment that offers the advantage of standardization across test sessions. By constructing the PEBL paradigm, we wished to induce a certain type of stress to healthy subjects which can easily be repeatable and is readily available since the software used can be downloaded free of charge. Different laboratory employed stress tasks are found to differ in their ability to reliably evoke HPAA responses [29]; thus, through a panel of salivary biomarkers, we decided to evaluate the capability of a PEBL test battery to activate HPAA and cause the changes in salivary total antioxidative capacity.

As mentioned, salivary oxidative/antioxidant status, apart from that it mimics the whole-body image, might play a crucial role in some oral cavity-related diseases [7]. In the oral cavity, the sources of free radicals can be periodontal inflammation, xenobiotics (ethanol, cigarette smoke, and drugs), food, dental treatment, and dental material (e.g., fixed orthodontic appliances) [30]. To the best of our knowledge, no publications are dealing with the changes in salivary oxidative status and “pure” and short-term acutely applied psychological stress. In a previous study, the salivary antioxidant status of children, estimated through a panel of different parameters, was found to be increased prior to a tennis completion, which was associated with psychological stress [31]. It was shown that an exam, as a stimulus for psychological stress, causes an increase in salivary antioxidant status, while diminishes the oxidant counterpart [11]. Also, blood oxidative status was found to positively correlate with the poorer cognitive processes in night-shift health care workers [32].

The herein employed method for the determination of the total antioxidant capacity, the DPPH methods, might not be fully sensitive to minor changes in the salivary antioxidant/oxidant capacity. The reason for this is directly related to the stability of the DPPH radical, which, in turn, needs a strong oxidant radical in order to be quenched [33]. These facts might explain the nonexisting changes in the total salivary antioxidant capacity that was measured post-PEBL test (Table 2). On the other hand, a previous study found that acute psychological stress induces an increase in salivary antioxidant enzymes (e.g., catalase) but does not increase salivary thiobarbituric reactive substances concentration [11]. Thus, the DPPH method might not completely be suitable for the detection of changes in the antioxidant/oxidant status of the saliva. Also, it might be that there are no significant alterations in this finely balanced system induced by the acute short-term psychological stressful stimulus presented in our study. Using a panel of bioassays, with higher sensitivity, which would determine more specific components of the antioxidant system (enzyme activities, reduced glutathione, uric acid, etc.) could perhaps pinpoint the exact molecular disturbance if existent.

Cortisol levels, both plasma (bound and free) and salivary, have a diurnal pattern, where the secretion is the highest in the morning, in some 50-75% of the population, and slowly decreases during the day, with the lowest levels usually occurring around midnight [34, 35]. Free cortisol detected in the saliva is found to perfectly correlate with the free blood cortisol which is responsible for the biological effects of the steroid hormone [15]. Short-term exposure to stressful stimuli releases cortisol which in the short terms may have adaptive effects since it leads to behavioral and physical changes associated with a stressor [36]. It takes roughly around 20 min for stressful stimuli to cause an increase in salivary cortisol levels [15]. The effects that cortisol exerts are mediated directly through the main brain structures, hippocampus, prefrontal cortex, etc., involved in stress processing and HPAA regulation [4, 37]. The response of HPAA and the release of cortisol as a response to psychological stressors is suggested to be more intense in males than females [15]. However, we think that our sample might not be sufficiently large enough as to segregate the results of different subjects based on their gender.

Since we collected saliva samples immediately after the PEBL test, which lasted roughly 30 min, cortisol levels might not have reached their maximal expected change since it takes around 20 min for cortisol from the serum to reach saliva [15]. However, we chose to collect the saliva at this time point in order to avoid any additional impact on the saliva content which might arise posttest. This is especially true for the levels of NO which could be rapidly changing due to different nonmental stress-related stimuli. Although we found that the PEBL tests double salivary cortisol levels (Table 2), the correlation with the change in the total antioxidant capacity and NO levels was negligible (Figure 2). On a previous occasion, a psychologically induced increase in salivary cortisol was not found to correlate with damaged salivary RNA, which was studied as a potential psychologically stress biomarker [38].

Although still not completely reliable for the measurement of stress intensity, measuring the changes in the salivary IgA levels found its purpose in estimating some “types” of stressful events, e.g., academic stress [39]. The levels of IgA vary depending on the period when the stress is occurring. It is found that IgA increased in days before an exam (test), while it decreased after the announcement of the scores; thus, it was interpreted to be associated with academic success [6, 39]. Some of the tests in the PEBL test battery that include the presentation of the accomplished results (score) between the test sessions might be considered a stimulus for the increase/improvement in their result, i.e., a tendency towards some kind of success. Thus, the results obtained in our study, a decrease in the salivary IgA after the PEBL test, follow the previously reported trend [39]. There are several explanations why the levels of salivary IgA, an index of mucosal immunity, are decreased following exposure to stress and one of them involves the influence of an increase in cortisol, a known immunosuppressant [40]. Supporting the findings of other studies [41, 42], we found that there is a statistically significant negative correlation between salivary IgA and cortisol levels (Figure 2).

Nitric oxide is one of the gaseous neurotransmitters which possesses numerous physiological and pathological roles; however, it is known to cause tissue molecules damage, especially proteins. It is present in the form of nitrates in numerous biological fluids, including saliva where its origin is mainly from the circulation since the salivary glands actively reabsorb nitrates from the circulation [43]. As in the case of cortisol, the action of this neurotransmitter is related to the function of various brain structures involved in stress processing [44]. The usage of this biomarker for the estimation of stress-induced biological response is still debatable. This is especially true for psychological stress, where salivary nitrates and daily psychological stress and anxiety were not found to be connected [45]. In the present study, a significant increase in the salivary nitrate concentrations was found following the PEBL test battery (Table 2), which displayed a weak negative correlation with the antioxidative capacity (Figure 3). These data could easily be explained by the fact that although NO might represent both oxidative and mental stress; hence, the absence of change in the total salivary antioxidative capacity and the observed negative correlation suggest that this increase might be solely from the mental stress. These results are in partial agreement with the obtained increase in salivary cortisol concentrations. Namely, the activity of HPAA and cortisol release are partially dependent on the circulating nitrates, which in turn relate to the salivary levels of the same [45].

Apart from the importance in the study of (acute) mental stress, salivary redox biomarkers found their application in different neurological and psychiatric studies. In patients suffering from stroke, salivary redox status was imbalanced, and a significant oxidative damage to proteins and lipids was established [46]. Also, in patients with dementia, salivary advanced glycation end products were found to correlate with the deterioration in mini mental state examination results, suggesting that this salivary parameter might be useful for noninvasive diagnosis of dementia [47].

## 5. Strengths and Limitations of the Study

One of the main limitations of this study is the sample structure itself, i.e., healthy young people in their late adolescence and early adulthood. On the other hand, stress is salient and omnipresent in young people's lives; thus, this particular sensitive group might be good for constructing a test battery to mimic stress. Young people are more sensitive to stress than older ones that ought to be adapted to stress and have built mechanisms on how to cope with it. Also, in young people, biological mechanisms that are affected by an acute stress situation might be more sensitive and better responsive than in adults. In addition, gender may have a significant influence on the obtained results, having in mind that two-thirds of the sample subjects were females. However, due to the relatively limited sample size, we were unable to divide and compare the results according to the subjects' gender. The method employed for the determination of the total antioxidant status might not be fully sensitive for the determination of minute changes occurring in oxidative stress under the psychological stressor. One of the major strengths of the study lays in the type of the investigated biological sample, saliva, used for the estimation of stress levels. The collection of saliva is relatively easy and represents a noninvasive technique which enables the acquisition of a valuable source that could be used for the determination of different biological parameters related to the response to stress. A recent review paper highlighted the importance of salivary alpha amylase activity as a valid and reliable stress biomarker that had emerged in behavioral medicine studies [48]. In our future studies we plan to investigate the connection between salivary alpha amylase activity and salivary oxidative stress parameters.

## 6. Conclusions

The obtained results indicate that the constructed PEBL test battery, consisting of 4 tests, might represent an adequate laboratory stress-inducing paradigm since it evokes biological response characterized by a significant increase in salivary cortisol and NO_2_^−^/NO_3_^−^ levels, while it leads to a decrease in IgA levels. On the other hand, although it induced the changes in stress-related biological parameters, it did not affect salivary antioxidative capacity. These results suggest that the acute short-term stress induced by the PEBL test battery here used does evoke the changes in the salivary mental stress-related parameters, but not in the total antioxidative capacity.

## Figures and Tables

**Table 1:** Study group characteristics.

Subjects characteristics (*n* = 36)		
	Mean ± SD	Median (IQR)
Age	20.7 ± 0.9	20 (20-21)
Body height (cm)	173.1 ± 12.7	170 (161-171)
Body weight (kg)	67.1 ± 13.8	60 (53.5-65)
Body mass index	22.4 ± 2.1	22.4 (20.3-23.0)
Subject gender	Male	Female
	12	24

IQR, interquartile range.

**Table 2:** Salivary biochemical parameters obtained from the subjects pre- and post-PEBL test.

	Mental stress-related parameters		Oxidative stress-related parameters	
	Cortisol (ng/mL)	IgA (ng/mg of proteins)	NO_2_^−^/NO_3_^−^ (*μ*mol/mg of proteins)	Antioxidative capacity (% of DPPH quenching)
Pre-PEBL				
Median	1.4	197.7	56.9	14.7
Max	3.1	245.7	262.3	16.6
Min	0.4	48.2	5.6	6.7
IQR	0.8–2.3	134.9–245.7	23.6–103.9	11.3–16.3
Post-PEBL				
Median	1.6	262.3	71.8	9.9
Max	3.9	81.7	418.0	18.8
Min	0.5	0.3	18.9	1.4
IQR	0.9–2.5	81.7–159.6	30.0–108.9	5.3–17.9
Percent change after test (%)	52.9	-37.9	61.1	0.9
*Z* value	-2.247	-4.745	-2.718	-0.966
*p* value	0.025	<0.001	0.007	>0.05
*r* value	0.375 (m)	0.791 (l)	0.453 (m)	0.161 (s)

IQR, interquartile range. Effect size estimate values were interpreted as follows: small, s; medium, m; large, l.

## Data Availability

Data available on request from the corresponding author.
